# Health Care Expenditures Among Individuals With Chronic Psychotic Disorders in Ontario: An Analysis Over Time

**DOI:** 10.3389/frhs.2022.848072

**Published:** 2022-03-21

**Authors:** Claire de Oliveira, Tomisin Iwajomo, Paul Kurdyak

**Affiliations:** ^1^Centre for Health Economics and Hull York Medical School, University of York, York, United Kingdom; ^2^Institute for Mental Health Policy Research and Campbell Family Mental Health Research Institute, Centre for Addiction and Mental Health, Toronto, ON, Canada; ^3^ICES, Toronto, ON, Canada; ^4^Institute of Health Policy, Management, and Evaluation, University of Toronto, Toronto, ON, Canada; ^5^Department of Psychiatry, Faculty of Medicine, University of Toronto, Toronto, ON, Canada; ^6^Mental Health and Addictions Centre of Excellence, Ontario Health, Toronto, ON, Canada

**Keywords:** psychosis, schizophrenia, health care expenditures, costs, time series

## Abstract

Chronic psychotic disorders are severe and disabling mental disorders associated with poor psychiatric and medical outcomes, and among the most costly mental disorders to treat. Understanding trends in aggregate health care expenditures over time, and respective drivers, can provide relevant insights for decision makers, namely around appropriate allocation of scarce resources within the health care sector. Using administrative health care times series data from Ontario, this analysis examined trends in aggregate public health care expenditures and activity from 2012 to 2019 among all individuals with a diagnosis of a chronic psychotic disorder. Total aggregate health care expenditures for individuals with a chronic psychotic disorder in Ontario increased at a moderate rate over this time period, in line with the growth of the number of people diagnosed, and thus not likely driven by unit costs or resource use. Psychiatric hospitalizations made up the largest share of health care expenditures (~30%). Nonetheless, among all health services, expenditures of acute medical hospitalizations, outpatient prescription drugs and home care saw the largest growth over time. Mean/per capita health care expenditures were greater for females, and increased with age as well as with the presence of comorbidities/chronic conditions. In particular, mean/per capita health care expenditures increased steadily with the number of comorbidities and were highest for individuals with 5 or more comorbidities and those with congestive heart failure, highlighting the ever-increasing importance of addressing physical health conditions among this patient population. These findings will have important implications for decision makers, namely around the appropriate allocation of health care resources for patients with chronic psychotic disorders. Future research should continue to monitor health care expenditures for individuals with chronic psychotic disorders as well as extend this analysis beyond 2019 to understand how the COVID-19 pandemic, and resulting lockdowns, has impacted aggregate health care expenditures and outcomes for patients living with chronic psychotic disorders.

## Introduction

Chronic psychotic disorders are severe and disabling mental disorders associated with poor psychiatric and medical outcomes. Despite affecting only 1–1.2% of the population, these disorders are associated with high health care costs due to the young age at onset and the need for intensive care over the life course (1). Understanding trends in health care expenditures over time, and respective drivers, can provide relevant insights for decision makers, namely around appropriate allocation of scarce resources within the health care sector. Unfortunately, little work has examined changes in aggregate health care expenditures for chronic psychotic disorders-related care over time.

In Ontario, Canada, the economic burden of chronic psychotic disorders to the public health care system was estimated to be roughly $2.1 billion CAD in 2012, the equivalent to 3% of the provincial health care budget for that year (1). It was also found that costs varied greatly by age groups and, in some instances, sex—while psychiatric hospitalizations accounted for most of the cost at younger ages, in particular for males (likely due to the earlier onset of the illness), long-term care and acute medical hospitalizations accounted for most of the cost for older age groups, due to increasing morbidity with age. Ultimately, these findings highlighted the need for health care systems to address both physical and mental illness simultaneously, especially for older patients with chronic psychotic disorders, and to understand how the interplay between mental and physical health contribute to increased costs among patients with chronic psychotic disorders. However, this work only examined data for 1 year and did not examine how/whether health service expenditures changed over time or whether they differed by patient profile, in particular morbidity. This analysis sought to examine trends in total aggregate public health care expenditures and activity in Ontario from 2012 to 2019 for individuals with chronic psychotic disorders to understand how expenditures reflect trends in activity and changes in patient profiles and morbidity.

## Materials and Methods

### Data

This analysis employed health care records housed at ICES (formerly known as the Institute for Clinical Evaluative Sciences) in Toronto, Ontario, and collected through the administration of Ontario's public health care insurance plan. These data include individual-level linkable and longitudinal data on most publicly funded health care services for all legal residents of Ontario, Canada's most populous province. Data on institution-based care are recorded in the Discharge Abstract Database (acute medical hospitalizations and psychiatric hospitalizations in non-psychiatric designated beds), the Ontario Mental Health Reporting System (psychiatric hospitalizations in psychiatric designated beds), the Continuing Care Reporting System (continuing and long-term care), and the National Rehabilitation Reporting System (rehabilitation); data on ambulatory care, such as emergency department visits, are included in the National Ambulatory Care Reporting System. The Ontario Health Insurance Plan claims database captures data on physician visits, including fee-for-service visits and shadow-billed services, as well as laboratory and diagnostic claims. The Ontario Drug Benefit Program claims database includes information on all outpatient prescription drugs dispensed to individuals covered under the provincial public drug plan (i.e., individuals aged 65 years and older, as well as those under the age of 65 years living in a long-term care home, a home for special care or a Community Home for Opportunity, receiving professional home and community care services, enrolled in the Trillium Drug Program, or on social assistance). The Home Care Database records all unique visits provided by home care professionals. The Registered Persons Database, a population-based registry, provides basic demographic data, such as age and sex, on all legal residents of Ontario and their eligibility for public health care insurance. A full description of these databases can be found in Supplementary Table 1. All databases were linked using unique encoded identifiers and analyzed at ICES, whose legal status under Ontario's health information privacy law allows it to collect and analyse demographic and health care data, without consent, for health system evaluation and improvement. The use of health care data in this project was authorized under section 45 of Ontario's Personal Health Information Protection Act.

To construct the times series, all individuals over the age of 15 eligible for public health care insurance ever diagnosed with chronic psychotic disorder were selected on January 1st of each year of the analysis, using a validated algorithm (2). More specifically, this included anyone who had a hospitalization with a diagnosis of schizophrenia, schizoaffective disorder, and psychosis not otherwise specified in the Discharge Abstract Database (using ICD-10 codes F20, excluding F20.4, F25, and F29) and the Ontario Mental Health Reporting System (using DSM-IV codes 295.^*^ and 298.^*^) since 1988, and/or 3 physician visits for psychosis-related care within a 3 year window in the Ontario Health Insurance Plan claims database since 1991. Patients with a diagnosis of psychotic disorder not otherwise specified were included in the analysis as evidence suggests that these patients are ultimately diagnosed with schizophrenia or schizoaffective disorder (3). Once all individuals who were ineligible for public health care insurance were dropped (~46% of all individuals ever diagnosed with psychosis), those not residing in the province over the analysis period as well as those under the age of 16 (as psychosis is quite rare before this age) and over the age of 105 (as any age >105 is likely an error) were further excluded (where these last 3 exclusions made up <1% of the remaining sample).

The Johns Hopkins Adjusted Clinical Groups (ACG)® System Version 10 software (4) was used to determine comorbidities, which were estimated through the use of proprietary software and hospitalization and physician billings data. Comorbidities were defined as the more limiting ACG® System Aggregated Diagnosis Group (ADG) categories: ADG 3—Time limited: Major, ADG 4—Time limited: Major—Primary Infections, ADG 9—Likely to Recur: Progressive, ADG 11—Chronic Medical: Unstable, ADG 16—Chronic Specialty: Unstable-Orthopedic, ADG 22—Injuries/Adverse Effects: Major, and ADG 32—Malignancy. The ADGs were calculated at the start of each calendar year using a 2-year look-back period. Chronic conditions were ascertained through existing ICES-derived cohorts and acquired registries, and included asthma [Asthma Database (5)], cancer [Ontario Cancer Registry (6)], chronic obstructive pulmonary disorder [COPD Database (7)], congestive heart failure [Congestive Heart Failure Database (8)], Crohn's/colitis [Ontario Crohn's and Colitis Cohort Database (9)], diabetes [Ontario Diabetes Database (10)], HIV [Ontario HIV Database (11)], hypertension [Ontario Hypertension Database (12)], and rheumatoid arthritis [Ontario Rheumatoid Arthritis Database (13)]. An overview of the patient profile in 2012 can be found in Supplementary Table 2.

### Analysis

#### Estimation of Health Care Expenditures

A validated cost algorithm, available at ICES, was employed to estimate all direct patient-level health care costs from the third-party public payer perspective (i.e., the Ontario Ministries of Health and Long-term Care) (14). The costing methodology defined in the algorithm uses a bottom-up/micro-costing approach to cost services at the individual patient level, which identifies individual episodes of care or utilization in the health care system and attaches costs or amounts paid (or where lacking, prices) to each one. Given Ontario's public health insurance system, prices are rarely set by providers in a private marketplace; therefore, costs/amounts paid by the Ministry of Health were used. In cases where individual unit costs were not available (e.g., long-term care), a top-down approach, which allocates corporate aggregate (i.e., institutional) costs to individual visits or cases/episodes of care, was employed. Further details on the costing methodology can be found elsewhere (14). The costs captured by the algorithm account for over 90% of costs of all government paid health care (15). Given the close correspondence between costs and expenditures, the measure of costs of episodes used is indicative of health care expenditures. Health care costs were categorized into the follow health care expenditure categories: acute medical hospitalizations, psychiatric hospitalizations, and other institution-based care (i.e., inpatient rehabilitation, complex continuing care, and long-term care), hospital outpatient clinic visits, emergency department (ED) visits, other ambulatory care (i.e., same-day surgery, cancer clinic visits, and dialysis clinic visits), physician services, outpatient prescription drugs, and home care. All costs were expressed in 2020 Canadian constant dollars.

#### Analysis of Annual Health Care Expenditures

One of the main concerns for health policy makers is to understand the drivers in the growth of aggregate health care expenditures. For example, it is important to understand whether expenditures change due to changes in the number of individuals diagnosed with a given condition and accessing health care services or whether the average person is sicker than before and thus using health care more intensively (either through the use of more services in general or using them for longer periods of time, e.g., longer hospitalizations) or whether care has become more costly (i.e., the price of health care has increased over time due to technological advancements).

To ascertain this, health care expenditure profiles (i.e., total and mean/per capita expenditures) were estimated annually from 2012 to 2019 and examined by sex and health service. In addition, total and mean/per capita health care expenditures were compared to health care activity profiles each year, namely the number of individuals receiving treatment and the mean number of episodes of care for the most costly health care encounters, acute medical and psychiatric hospitalizations, as well as the respective mean length of stay. Finally, annual health care expenditures were examined by age and morbidity profiles (i.e., mean/per capita expenditures per number of co-morbidities and per chronic condition). Linear regression models with number of patients/cost as the dependent variables and year as the independent variable were estimated to determine the significance of trends over time. For each slope estimated, the respective *p*-value was estimated.

## Results

### Health Care Expenditure Profiles by Year

**Figure 1:**
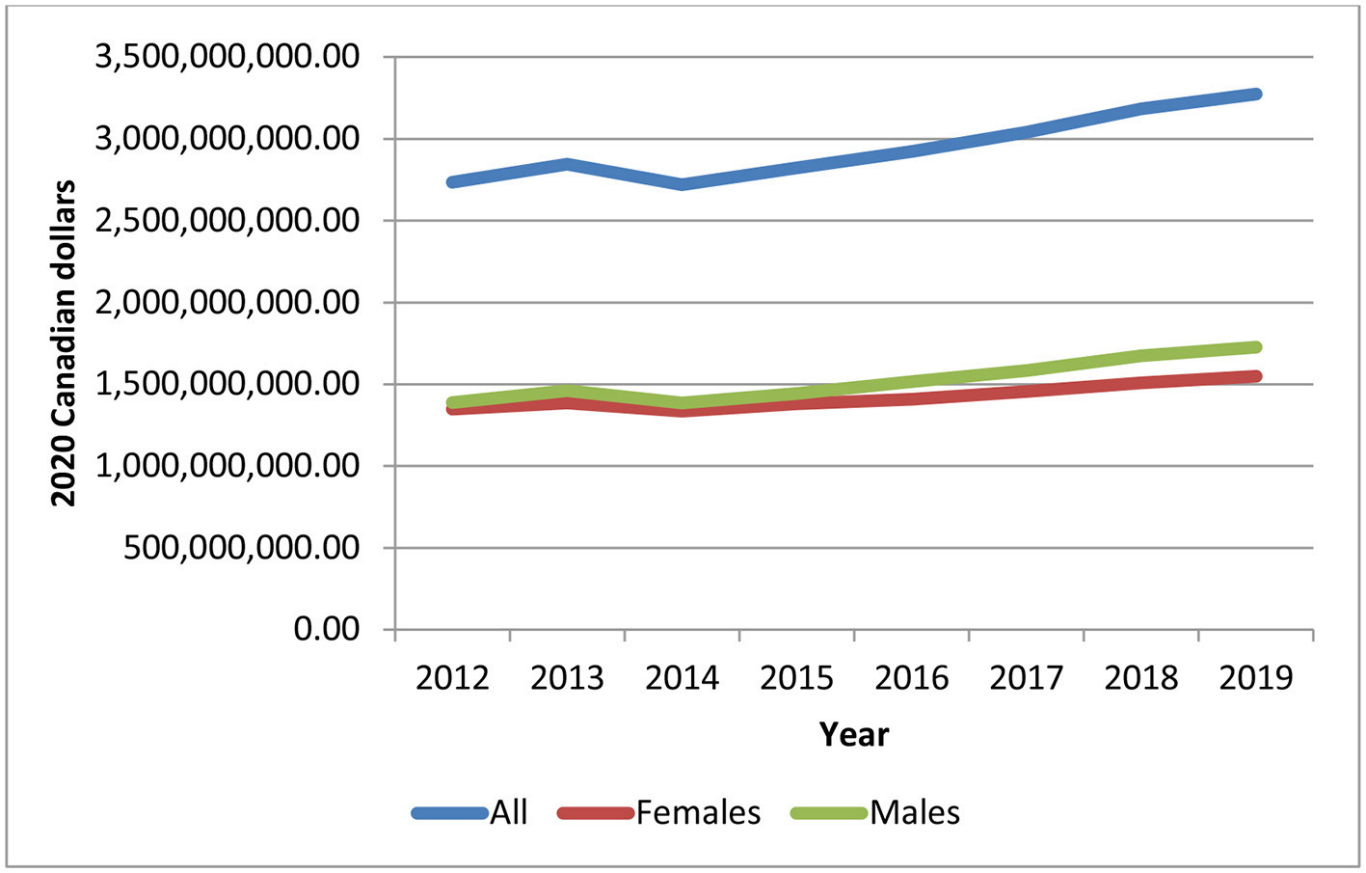
Total health care expenditures for individuals diagnosed with chronic psychotic disorders, 2012-2019, all and by sex. Source: administrative health care data from ICES. The *p*-values of the trend analysis for all expenditures and expenditures for females and males are as follows: *p* = 0.0002, *p* = 0.0012, and *p* = 0.0008, respectively).

**Figure 2:**
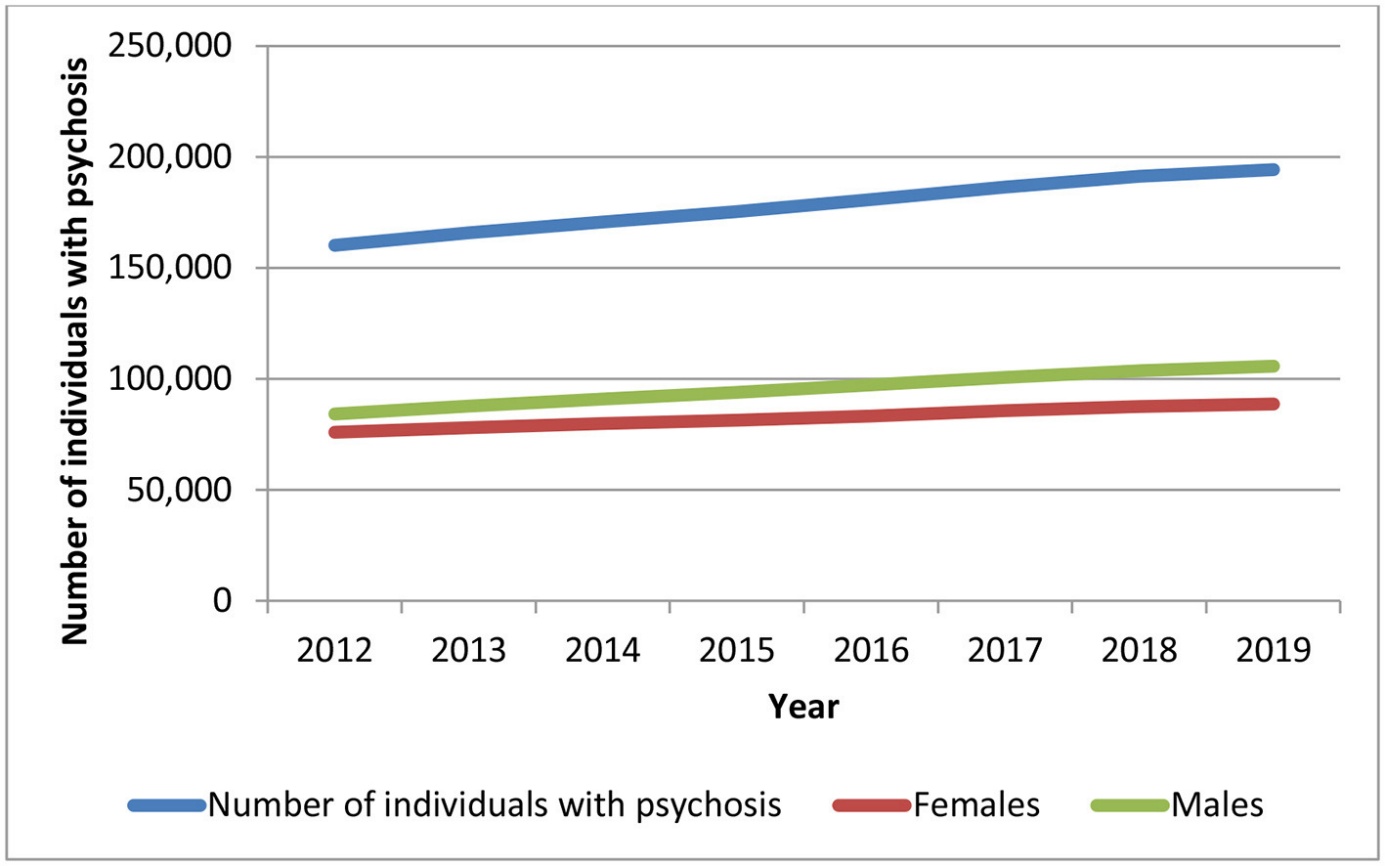
Number of individuals with a chronic psychotic disorder in Ontario, 2012–2019, all and by sex. Source: administrative health care data from ICES. The *p*-values of the trend analysis for all expenditures and expenditures for females and males are as follows: *p* = 0.0064, *p* < 0.0001, and *p* < 0.0001, respectively); the *p*-value for the differences between females and males is *p* < 0.0001.

**Figure 3:**
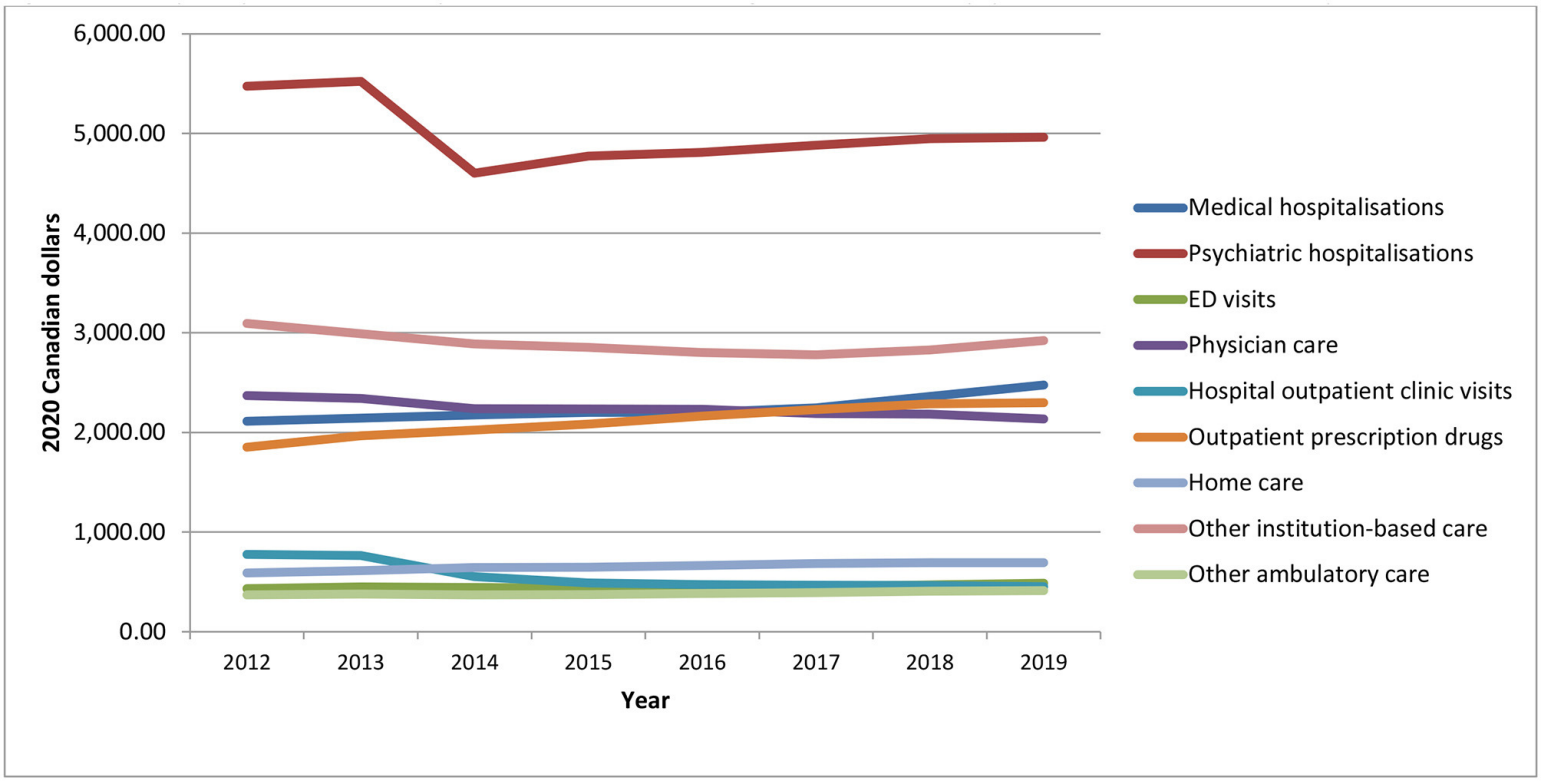
Mean/per capita health care expenditures for individuals diagnosed with chronic psychotic disorders, 2012–2019, by health service. Source: administrative health care data from ICES. The *p*-values from the trend analysis for mean/per capita expenditures are as follows: medical hospitalizations, *p* = 0.0011; psychiatric hospitalizations, *p* = 0.214; ED visits, *p* = 0.0093; physician care, *p* = 0.0003; hospital outpatient clinic visits, *p* = 0.0059; outpatient prescription drugs, *p* < 0.001; home care, *p* < 0.001; other institution-based care, *p* = 0.0747; other ambulatory care, *p* = 0.0013.

Despite some variation between 2012 and 2014, total aggregate health care expenditures for individuals with chronic psychotic disorders increased at a steady rate over time (Figure 1). On average, total health care expenditures increased 19.7% from 2012 to 2019, from $2.7 billion in 2012 to about $3.3 billion in 2019 (in 2020 Canadian dollars) (*p* = 0.0002). Health care expenditures increased over time for both sexes (males, *p* = 0.0008; females, *p* = 0.0012), but were slightly higher for males for all years, though the difference between sexes increased slightly in more recent years. The number of individuals living with chronic psychotic disorders rose steadily from 160,195 individuals in 2012 to 194,335 individuals in 2019 (all, *p* = 0.0064; males, *p* < 0.0001; females, *p* < 0.0001) (Figure 2). There were slightly more males than females with chronic psychotic disorders (*p* < 0001). Mean/per capita expenditures were relatively constant over time (varying between $16,000 and $17,000), with a slight dip in 2014 (Supplementary Figure 1) and were greater for females compared to males. Broken by type of health service, total health care expenditures were highest for psychiatric hospitalizations, which were about $878 million in 2012, decreasing to $785 million in 2014, and growing slowly until 2019, reaching about $964 million. However, the largest increases in health care expenditures over time were observed for acute medical hospitalizations, outpatient prescription drugs and home care, which increased 51, 42, and 42% (*p* = 0.0011; *p* < 0.0001; *p* < 0.0001), respectively, from 2012 to 2019 (Supplementary Figure 2). These trends also held for mean/per capita expenditures (Figure 3), where the majority of health care expenditures were due to psychiatric hospitalizations (~30%), followed by other institution-based care (~17%), acute medical hospitalizations (~12–15%), physician services (~13–14%), and outpatient prescription drugs (~11–14%).

### Health Care Activity Profiles by Year

**Figure 4:**
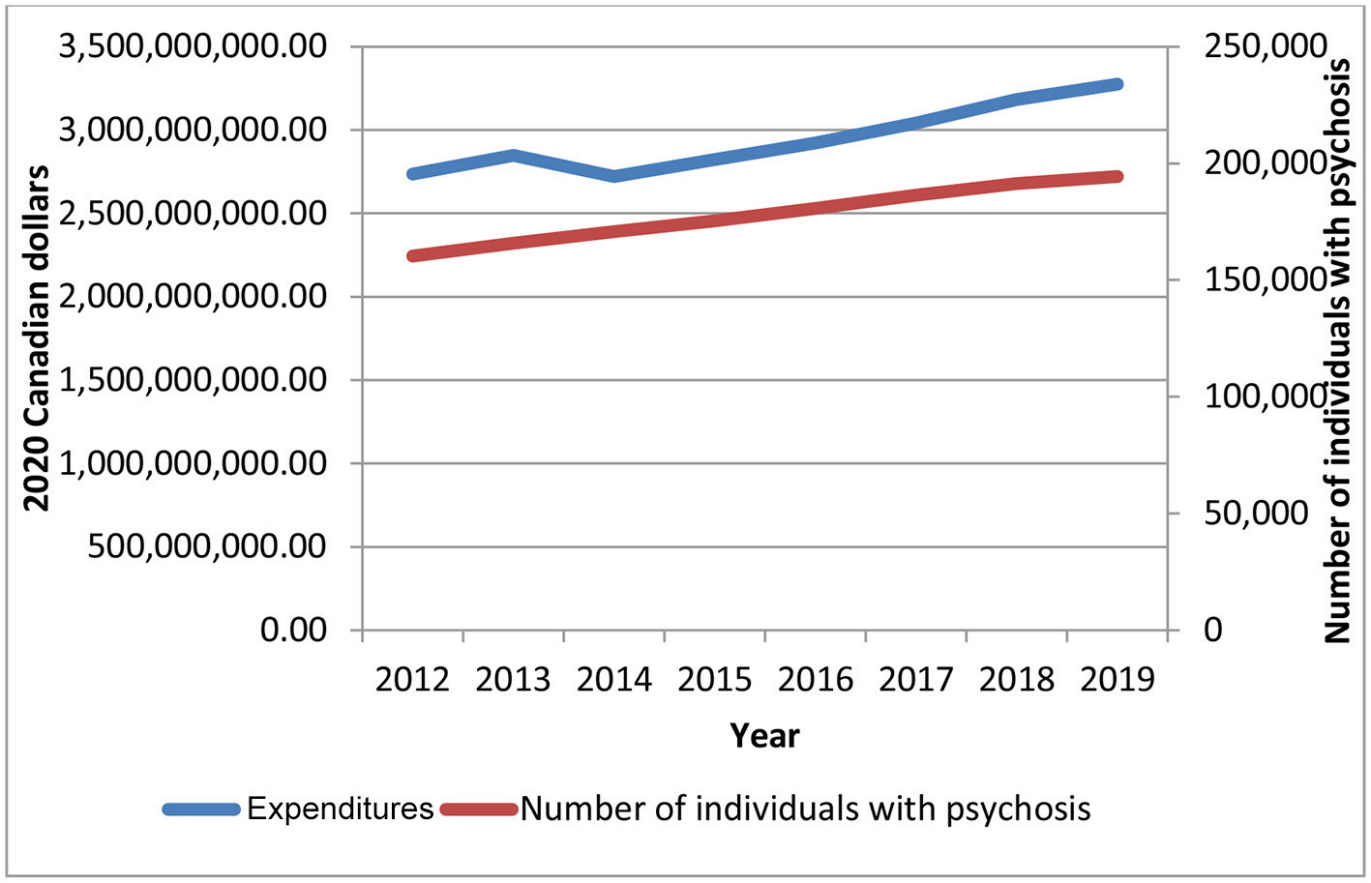
Total health care expenditures and number of individuals with a chronic psychotic disorder in Ontario, 2012–2019. Source: administrative health care data from ICES. The *p*-values from the trend analysis for total expenditures and number of individuals with a chronic psychotic disorder are as follows: *p* = 0.0002 and *p* = 0.0064.

To understand the evolution of mean/per capita expenditures over time, it is important to jointly examine total health expenditures and total number of individuals with chronic psychotic disorders as well as the changes in health activity, where applicable. While total aggregate health care expenditures increased by 19.7% (*p* = 0.0002), the total number of prevalent cases increased at a similar rate, 21.3% (*p* = 0.0064) (Figure 4). The mean number of psychiatric hospitalizations from 2021 to 2019 remained relatively stable over time (at about 1.7 episodes per patient) (Supplementary Figure 3); these were slightly higher among females than males. The average length of stay of these hospitalizations also remained relatively constant over time (roughly 12–13 days) and, although similar between males and females, was slightly higher for females (Supplementary Figure 4). The number of acute medical hospitalizations was also relatively stable over time (1.5–1.6 episodes per patient) but was slightly higher for males than females (Supplementary Figure 5), while the average length of stay increased slightly over time (from 15 to 17 days) and was higher for males (Supplementary Figure 6).

### Health Care Expenditures by Age

**Figure 5:**
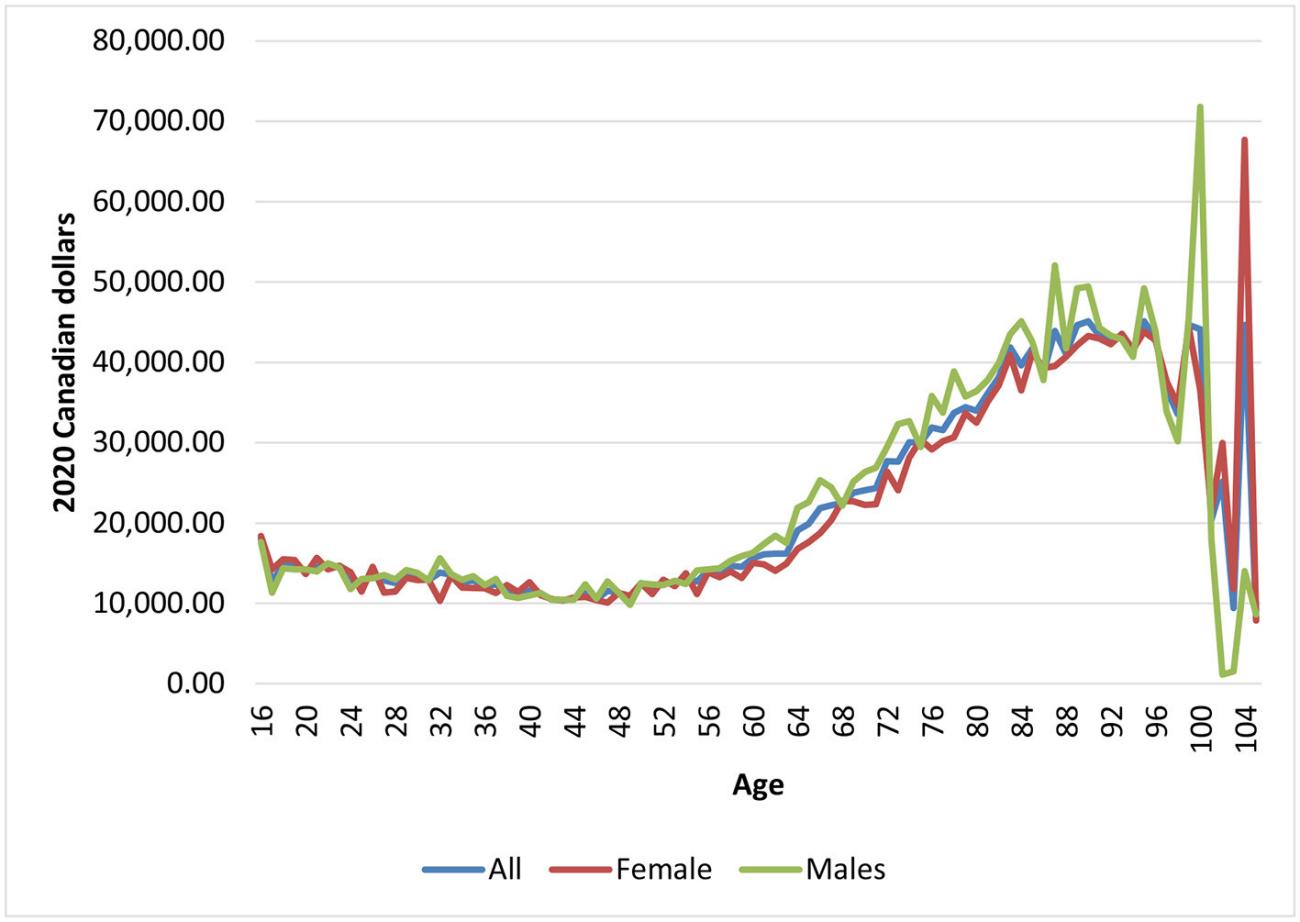
Mean health care expenditures for individuals diagnosed with chronic psychotic disorders, 2012–2019, by age and sex. Source: administrative health care data from ICES.

Figure 5 provides the mean/per capita health care expenditures by age in 2019, the last year of the analysis. Overall, health care expenditures followed an s-shaped/sigmoidal curve from 16 to 105 years old. Mean/per capita expenditures were roughly $18,000 at age 16 (typically the age of first episode psychosis) and then decreased to just under $10,500 at age 43. Not surprising, mean/per capita expenditures then increased with age, due to higher expenditures of acute medical hospitalizations and other institution-based care (not shown), peaking at just under $42,000 around age 83 (Figure 5) (expenditures at older ages varied quite a bit due to smaller sample sizes). On average, males had higher costs than females across ages, in particular at older ages.

### Morbidity Profiles by Year

**Figure 6:**
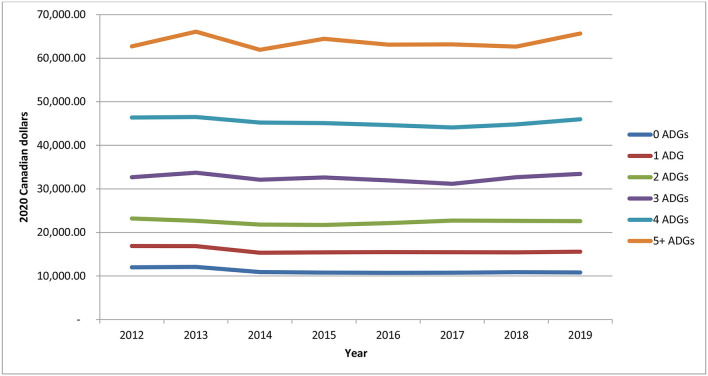
Mean/per capita health care expenditures for individuals diagnosed with chronic psychotic disorders, 2012–2019, by number of comorbidities (measured using the Johns Hopkins Aggregated Diagnosis Groups software). Source: administrative health care data from ICES. The *p*-values of the trends analysis for mean/per capita expenditures are as follows: 0 ADGs, *p* = 0.03; 1 ADG, *p* = 0.0537; 2 ADGs, *p* = 0.897; 3 ADGs, *p* = 0.7734; 4 ADGs, *p* = 0.1959; 5+ ADGs, *p* = 0.7856.

**Figure 7:**
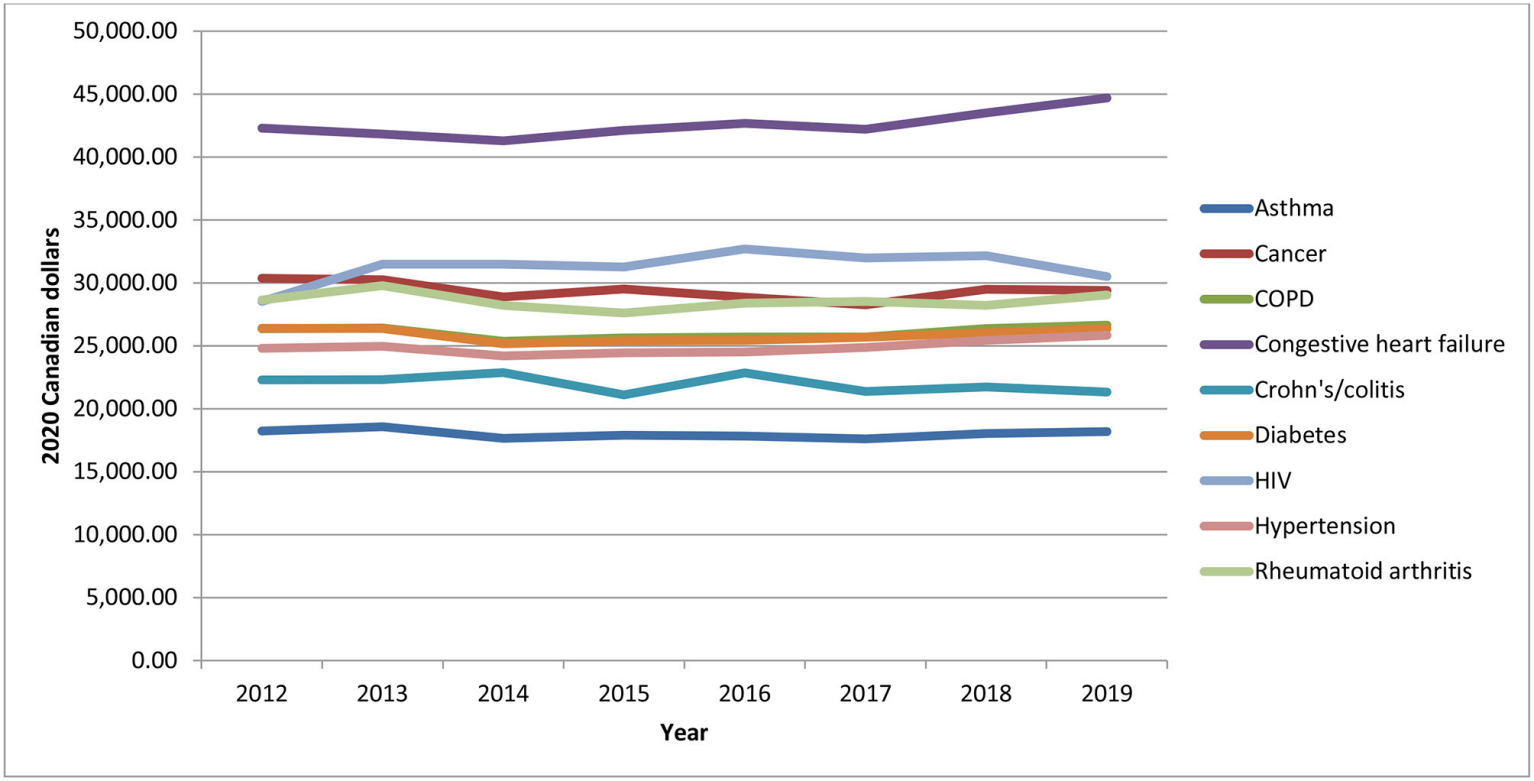
Mean/per capita health care expenditures for individuals diagnosed with chronic psychotic disorders, 2012–2019, by chronic condition. Source: administrative health care data from ICES. The *p*-values of the trends analysis for mean/per capita expenditures are as follows: asthma, *p* = 0.4995; cancer, *p* = 0.1721; COPD, *p* = 0.6726; congestive heart failure, *p* = 0.023; Crohn's/colitis, *p* = 0.1878; diabetes, *p* = 0.9866; HIV, *p* = 0.2584; hypertension, *p* = 0.0928; rheumatoid arthritis, *p* = 7,172.

From 2012 to 2019, on average, each individual had at least one comorbidity/chronic condition, with females having slightly more than males (Supplementary Figures 7, 8). Although the changes over time were quite small, the number of comorbidities and chronic conditions has been increasing slightly over time, which is in line with the increases seen in expenditures of acute medical hospitalizations. Total health care expenditures were higher for individuals with no comorbidities, due to the larger number of healthier individuals living with a chronic psychotic disorder (Supplementary Figure 9). Nonetheless, expenditures generally increased over time for all groups (though expenditures were somewhat constant over time for individuals without comorbidities). Figure 6 depicts the mean/per capita expenditures from 2012 to 2019 by number of comorbidities, where mean/per capita health care expenditures increased with the number of comorbidities. However, mean/per capital health care expenditures remained fairly constant over time (*p*-values for trends for 0 to 5+ comorbidities were as follows: *p* = 0.03, *p* = 0.0537, *p* = 0.897, *p* = 0.7734, *p* = 0.1959, *p* = 0.7856, respectively). Individuals without comorbidities had mean/per capita expenditures of about $11,000–$12,000, while those with five or more comorbidities had expenditures between $62,000 and $65,000. Total health care expenditures were also higher for individuals with hypertension, diabetes, COPD, and asthma, all chronic conditions, which affect large numbers of individuals; furthermore, total expenditures for these chronic conditions saw the largest increases over the analysis period (Supplementary Figure 10). Mean/per capita expenditures also varied by chronic condition, but mostly remained constant over time, as shown in Figure 7 (where p-values varies between 0.03 and 0.9866). However, individuals with chronic psychotic disorders and congestive heart failure had the largest mean/per capita health care expenditures at $42,000–$45,000 and witnessed the largest increase over time.

## Discussion

The level of and changes in aggregate health care expenditures is a key concern for policy makers as health care expenditures make up a sizeable portion of government budgets. From 2012 to 2019, total aggregate health care expenditures of individuals with chronic psychotic disorders in Ontario increased at a moderate rate, in line with the number of patients living with a chronic psychotic disorder. However, health care expenditures by type of health service grew at different rates. While total expenditures of psychiatric hospitalizations increased by 10% from 2012 to 2019, total expenditures for acute medical hospitalizations, outpatient prescription drugs and home care increased by 51, 42, and 42% respectively, for the same time period. Moreover, there was some redistribution of costs from 2012 to 2019, where mean expenditures of psychiatric hospitalizations decreased slightly from 32.1 to 29.5% within total mean health care expenditures, while mean expenditures of acute medical hospitalizations and outpatient prescription drug increased from 12.4 to 14.7% and from 10.8 to 13.6%, respectively. Females typically had higher mean expenditures, as well as older individuals (as expenditures increased with age), individuals with multimorbidity (in particular, those with more than five comorbidities) and individuals with congestive heart failure.

Previous research from Australia found little change in public health care expenditures for individuals with psychosis over a 10-year period (16). Instead, the authors found a significant redistribution of expenditures within the public health care sector over time. In particular, they found that inpatient expenditures decreased from 78 to 42% of total health sector costs from 2000 to 2010, while expenditures with ambulatory care (including outpatient and community mental health care) and pharmaceutical treatment almost tripled over the same time period, this last driven by an increase in expenditures of atypical antipsychotic drugs (16). The rise over time in health care expenditures on anti-psychotic drugs has also been noted in the UK (17). These findings are generally in line with results of this analysis. For example, the decrease in expenditures for psychiatric hospitalizations over time, alongside the increase in expenditures of outpatient prescription drugs during the same time period, is likely due to the growing trend in Ontario of treating these individuals outside hospital settings.

In tandem, the increase in acute medical hospitalizations expenditures over time can be explained by the increasing comorbidity individuals experience as they age, as found elsewhere. Other work has shown that individuals with more comorbidities have higher expenditures (18). Research from Ontario has found that the presence of multiple chronic conditions was quite common among high-cost patients with severe mental illness, highlighting the need for quality of care interventions directed at managing psychosis and multimorbidity (19). The results of this analysis are in line with these findings, highlighting the need to address multimorbidity among patients with chronic psychotic disorders, as chronic physical health conditions are important drivers of health care spending. These findings show that individuals with cardiovascular disease, such as congestive heart failure, have higher mean health care expenditures compared to individuals with other chronic conditions, such as asthma. Patients with severe mental illness, such as psychosis, are less likely to receive standard levels of care for physical diseases, such as diabetes (20), as well as cardiovascular screening and prevention, when compared to a non-psychiatric population (21–23). Moreover, prior work has shown that the leading cause of death for individuals with chronic psychotic disorders is circulatory conditions, whereas for the general population it is cancer (24), suggesting that individuals with chronic psychotic disorders have not benefited from reductions in cardiovascular deaths to the same extent as the general population (25, 26).

These results have important implications for health policy makers as it shows that expenditures have largely increased as a function of the number of patients treated and less so due to increases in unit prices or resources (as evidenced, for example, by the relatively stable number of mean hospitalizations and length of stays over time). This work also provides some insight regarding the appropriate allocation of resources within the health care system for patients living with chronic psychotic disorders. Although not directly examined, on one hand, with further moves toward de-institutionalization (as suggested by the decrease in psychiatric hospitalization expenditures), more resources should likely be allocated to the provision of outpatient care, in particular psychotherapy, which is currently not covered under the Ontario Health Insurance Plan. On the other hand, given the high economic burden of chronic conditions and comorbidities, as evidenced in this analysis, more resources should also be invested in preventive health interventions to reduce the prevalence of obesity, diabetes, and smoking, which tend to be prevalent among these individuals and are predictive of the development of cardiovascular disease, such as congestive heart failure. Prior work has shown that patients with physical and mental co-morbidities are often ill served with potentially severe consequences (1). Finally, this work also provides important insights regarding cost differences by sex and the need for tailored approaches for females and males.

### Strengths and Limitations

The main contribution to the literature is the analysis of a long time series from a large Canadian province to examine trends in aggregate public health care expenditures among individuals with chronic psychotic disorders, overall and by sex, age, and morbidity. Most studies tend to examine hospital-based samples only (27); by including individuals who were diagnosed in outpatient settings in addition to hospital settings, this study included a sample representative of the entire population of individuals with a diagnosis of a chronic psychotic disorder. This analysis examined prevalent cohorts over multiple years and made use of the same databases for all years, thus enabling a direct comparison of expenditures over time. Finally, this work examined expenditures for several multimorbidity and chronic condition sub-groups, thus shedding light on the interaction between physical and mental health and its impact on health care expenditures.

The health care expenditures examined in this analysis account for most health care costs paid under a universal health care system. However, these expenditures do not include those of community care, which includes addiction services, and thus are an underestimate of the true health care spending for this patient population. Although psychiatric inpatient care is the mainstay for individuals with severe forms of psychosis, many patients make use of community care, the use of which has likely increased over the last decade in line with research done elsewhere (16). In addition, although in line with the perspective of this analysis, this analysis only examined outpatient drug expenditures for patients covered under the provincial public insurance plan. Finally, this analysis was done using data up to 2019 and thus did not examine the impact of the COVID-19 pandemic, and related lockdowns, on health care utilization and expenditures for this patient population. It will be important to understand how care has changed due to the pandemic and in particular how the uptake of telepsychiatry has changed health care utilization patterns and outcomes. Unfortunately, the impact of the pandemic on changes in mental health care delivery and utilization in this patient population, and related expenditures, has yet to be examined (28).

## Conclusion

Total aggregate health care expenditures for individuals with a chronic psychotic disorder in Ontario have been increasing at a moderate rate over time, in line with the growth of the number of people living with a chronic psychotic disorder. Although expenditures of psychiatric hospitalizations made up most of total expenditures, expenditures of acute medical hospitalizations, outpatient prescription drugs and home care have grown the most over the last 8 years. Mean/per capita health care expenditures were greater for females, and increased with age as well as with the presence of comorbidities/chronic conditions. In particular, mean/per capita health care expenditures were highest for individuals with five or more comorbidities and those with congestive heart failure, highlighting the ever-increasing importance of addressing physical health conditions among this patient population.

Future research should continue to monitor aggregate health care expenditures for individuals with chronic psychotic disorders but also explore the use of decomposition techniques to better understand changes in expenditures over time. Moreover, future work should seek to extend this analysis beyond 2019 to understand how the COVID-19 pandemic, and resulting lockdowns, has impacted health care utilization, and consequently health care expenditures, as well as outcomes for patients living with chronic psychotic disorders.

## Data Availability Statement

The data analyzed in this study is subject to the following licenses/restrictions: the dataset from this study is held securely in coded form at ICES. While legal data sharing agreements between ICES and data providers (e.g., healthcare organizations and government) prohibit ICES from making the dataset publicly available, access may be granted to those who meet pre-specified criteria for confidential access, available at www.ices.on.ca/DAS (email: das@ices.on.ca). The full dataset creation plan and underlying analytic code are available from the authors upon request, understanding that the computer programs may rely upon coding templates or macros that are unique to ICES and are therefore either inaccessible or may require modification. Requests to access these datasets should be directed to das@ices.on.ca.

## Author Contributions

CdO and PK conceptualized and designed the analysis. TI had access to the data and carried out the analysis, supervised by CdO. CdO drafted the initial manuscript and all authors critically reviewed the manuscript for important intellectual content. All authors interpreted the results and approved the final manuscript as submitted and agree to be accountable for all aspects of the work.

## Funding

This study was funded by the Canadian Institutes of Health Research and supported by ICES, which is funded by an annual grant from the Ontario Ministry of Health and Ministry of Long-term Care. The funders had no role in the design and conduct of the study; collection, management, analysis, and interpretation of the data; preparation, review, or approval of the manuscript; and decision to submit the manuscript for publication. The opinions, results, and conclusions reported in this article are also independent from the other sources that provided data and funding. No endorsement by ICES, the Ontario Ministry of Health and Ministry of Long-term Care is intended or should be inferred. Furthermore, parts of this material are based on data and/or information compiled and provided by the Canadian Institute for Health Information (CIHI) and the Ministry of Health. However, the analyses, conclusions, opinions, and statements expressed in the material are those of the authors and not necessarily those of CIHI or the Ministry of Health. In addition, parts of this material are based on data and information provided by Ontario Health (Cancer Care Ontario) (OH [CCO]). The opinions, results, view, and conclusions reported in this article were those of the authors and do not necessarily reflect those of OH (CCO). No endorsement by OH (CCO) was intended or should be inferred.

## Conflict of Interest

The authors declare that the research was conducted in the absence of any commercial or financial relationships that could be construed as a potential conflict of interest.

## Publisher's Note

All claims expressed in this article are solely those of the authors and do not necessarily represent those of their affiliated organizations, or those of the publisher, the editors and the reviewers. Any product that may be evaluated in this article, or claim that may be made by its manufacturer, is not guaranteed or endorsed by the publisher.
