# Supplementary material for: Health Care Expenditures Among Individuals With Chronic Psychotic Disorders in Ontario: An Analysis Over Time
Source: Front Health Serv. 2022 Mar 21;2:848072. doi: 10.3389/frhs.2022.848072 (PMC10012663; doi:10.3389/frhs.2022.848072)
Supplement: Supplementary file 1 [file Data_Sheet_1.pdf]

Appendix

Table A1. Administrative health care databases

Database	Setting	Description
Discharge Abstract Database	acute care hospitalisations	The Discharge Abstract Database is a national database, which contains demographic and clinical data on all acute care inpatient hospitalisations. It also includes data on psychiatric inpatient hospitalisations for children and adolescents and psychiatric inpatient hospitalisations, which occur in non-psychiatric designated beds.
Ontario Mental Health Reporting System	psychiatric hospitalisations	The Ontario Mental Health Reporting System collects demographic and clinical data on all adult psychiatric inpatient hospitalisations in Ontario.
Continuing Care Reporting System	complex continuing care, long-term care	The Continuing Care Reporting System contains demographic and clinical information on individuals receiving facility-based continuing care. These services include medical long-term care, rehabilitation, geriatric assessment, respite palliative care, and nursing home care.
National Rehabilitation Reporting System	rehabilitation	The National Rehabilitation Reporting System contains national data on rehabilitation facilities and clients, collected from participating adult inpatient rehabilitation facilities and programs.
National Ambulatory Care Reporting System	emergency department visits, same-day surgery and outpatient clinic visits	The National Ambulatory Care Reporting System contains data on all ambulatory care including emergency department visits, day surgery and outpatient clinic visits (for example, chemotherapy and dialysis).
Ontario Health Insurance Plan Claims Database	physician and outpatient services	The Ontario Health Insurance Plan Claims Database covers all services and procedures provided by health care providers who can claim under the

		Ontario Health Insurance Plan (such as, physician and laboratory/diagnostic services).
Ontario Drug Benefit Claims Database	outpatient prescription drugs	The Ontario Drug Benefit Claims Database includes data on all drugs dispensed in community pharmacies and long-term care/nursing facilities. The Ontario Drug Benefit program covers prescription drugs listed in the provincial formulary for all seniors (aged 65 and over) as well as those under the age of 65 living in a long-term care home, a home for special care or a Community Home for Opportunity, receiving professional home and community care services, enrolled in the Trillium Drug Program, or on social assistance.
Home Care Database	home care	The Home Care Database provides data on government-funded services coordinated by Ontario's Community Care Access Centres for individuals requiring home care.
Registered Persons Database	---	The Registered Persons Database is a population-based registry for Ontario and contains information on persons registered under the Ontario Health Insurance Plan.

Table A2. Overview of patient profile in 2012

	Females	Males	Total
	75,955	84,240	160,195
**Age**			
Mean ± SD	53.92 ± 18.18	46.49 ± 16.94	50.02 ± 17.93
Median (IQR)	53 (41-66)	46 (33-57)	49 (36-61)
**Neighbourhood income quintile**			
1 – low	23,650 (31.3%)	27,458 (32.9%)	51,108 (32.2%)
2 – medium low	16,139 (21.4%)	18,008 (21.6%)	34,147 (21.5%)
3 – medium	13,159 (17.4%)	14,291 (17.1%)	27,450 (17.3%)
4 – medium high	11,981 (15.9%)	12,848 (15.4%)	24,829 (15.6%)
5 – high	10,541 (14.0%)	10,754 (12.9%)	21,295 (13.4%)
**Rurality**			
No	68,871 (90.7%)	76,516 (90.9%)	145,387 (90.8%)
Yes	7,063 (9.3%)	7,689 (9.1%)	14,752 (9.2%)
**Local Health Integration Network**			
Erie St. Clair	3,925 (5.2%)	4,305 (5.1%)	8,230 (5.1%)
South West	5,798 (7.6%)	6,509 (7.7%)	12,307 (7.7%)
Waterloo Wellington	4,146 (5.5%)	4,269 (5.1%)	8,415 (5.3%)
Hamilton Niagara Haldimand Brant	9,361 (12.3%)	10,081 (12.0%)	19,442 (12.1%)
Central West	3,745 (4.9%)	4,174 (5.0%)	7,919 (4.9%)
Mississauga Halton	4,895 (6.4%)	5,093 (6.0%)	9,988 (6.2%)
Toronto Central	10,263 (13.5%)	12,734 (15.1%)	22,997 (14.4%)
Central	8,207 (10.8%)	8,402 (10.0%)	16,609 (10.4%)
Central East	8,324 (11.0%)	8,781 (10.4%)	17,105 (10.7%)
South East	2,795 (3.7%)	3,242 (3.8%)	6,037 (3.8%)
Champlain	6,870 (9.0%)	7,842 (9.3%)	14,712 (9.2%)
North Simcoe Muskoka	2,402 (3.2%)	2,802 (3.3%)	5,204 (3.2%)
North East	3,776 (5.0%)	4,241 (5.0%)	8,017 (5.0%)
North West	1,448 (1.9%)	1,765 (2.1%)	3,213 (2.0%)
**Number of psychiatric hospitalisations**			
Mean ± SD	1.72 ± 1.46	1.67 ± 1.28	1.69 ± 1.36
Median (IQR)	1 (1-2)	1 (1-2)	1 (1-2)
**Number of acute medical hospitalisations**			
Mean ± SD	1.48 ± 1.12	1.55 ± 1.27	1.51 ± 1.19
Median (IQR)	1 (1-2)	1 (1-2)	1 (1-2)
**Length of stay (days) for psychiatric hospitalisations**			
Mean ± SD	12.97 ± 25.90	11.58 ± 22.84	12.28 ± 24.43
Median (IQR)	5 (2-13)	4 (1-12)	4 (2-13)

**Length of stay (days) for acute medical hospitalisations**			
Mean ± SD	13.73 ± 24.30	16.30 ± 28.91	14.87 ± 26.49
Median (IQR)	5 (2-14)	6 (3-17)	6 (2-16)
**Comorbidities (measured by medical ADGs)**			
ADG 3-Time Limited: Major	8,126 (10.7%)	6,468 (7.7%)	14,594 (9.1%)
ADG 4-Time Limited: Major-Primary Infections	9,621 (12.7%)	10,339 (12.3%)	19,960 (12.5%)
ADG 9-Likely to Recur: Progressive	4,046 (5.3%)	3,784 (4.5%)	7,830 (4.9%)
ADG 11-Chronic Medical: Unstable	20,646 (27.2%)	17,705 (21.0%)	38,351 (23.9%)
ADG 16-Chronic Specialty: Unstable-Orthopedic	1,897 (2.5%)	1,618 (1.9%)	3,515 (2.2%)
ADG 22-Injuries/Adverse Effects: Major	16,967 (22.3%)	18,269 (21.7%)	35,236 (22.0%)
ADG 32-Malignancy	5,769 (7.6%)	4,875 (5.8%)	10,644 (6.6%)
**Chronic conditions**			
Asthma	15,931 (21.0%)	12,990 (15.4%)	28,921 (18.1%)
Cancer	5,410 (7.1%)	3,653 (4.3%)	9,063 (5.7%)
Congestive heart failure	4,316 (5.7%)	3,261 (3.9%)	7,577 (4.7%)
COPD	13,283 (17.5%)	11,806 (14.0%)	25,089 (15.7%)
Crohn's/colitis	658 (0.9%)	645 (0.8%)	1,303 (0.8%)
Diabetes	16,793 (22.1%)	14,759 (17.5%)	31,552 (19.7%)
HIV	156 (0.2%)	563 (0.7%)	719 (0.4%)
Hypertension	27,475 (36.2%)	22,280 (26.4%)	49,755 (31.1%)
Rheumatoid arthritis	1,252 (1.6%)	422 (0.5%)	1,674 (1.0%)
**Number of medical ADGs per person**			
Mean ± SD	0.88 ± 1.13	0.75 ± 1.07	0.81 ± 1.10
Median (IQR)	1 (0-1)	0 (0-1)	0 (0-1)
**Number of chronic conditions per person**			
Mean ± SD	1.12 ± 1.19	0.84 ± 1.06	0.97 ± 1.13
Median (IQR)	1 (0-2)	1 (0-1)	1 (0-2)
**Number of deaths**	2,296 (3.0%)	1,977 (2.3%)	4,273 (2.7%)

Legend: SD – standard deviation; IQR – interquartile range; ADG – aggregate diagnosis group; COPD – chronic obstructive pulmonary disorder; HIV – human immunodeficiency virus

Figure A1. Mean/per capita health care expenditures for individuals diagnosed with chronic psychotic disorders, 2012-2019, all and by sex

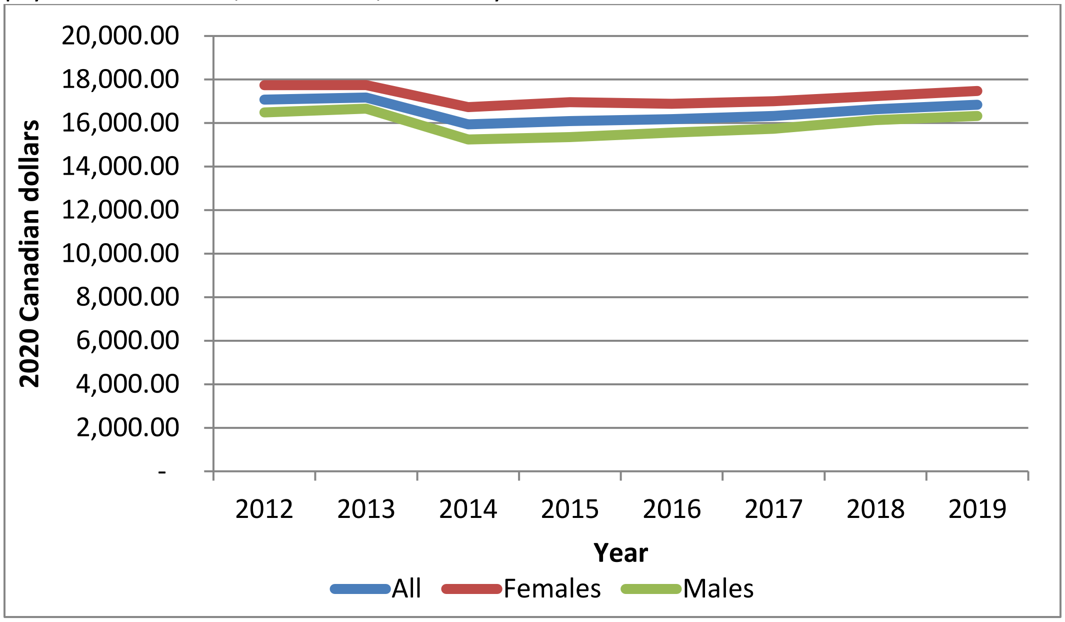

Source: administrative health care data from ICES

Figure A2. Total health care expenditures for individuals diagnosed with chronic psychotic disorders, 2012-2019, by health service

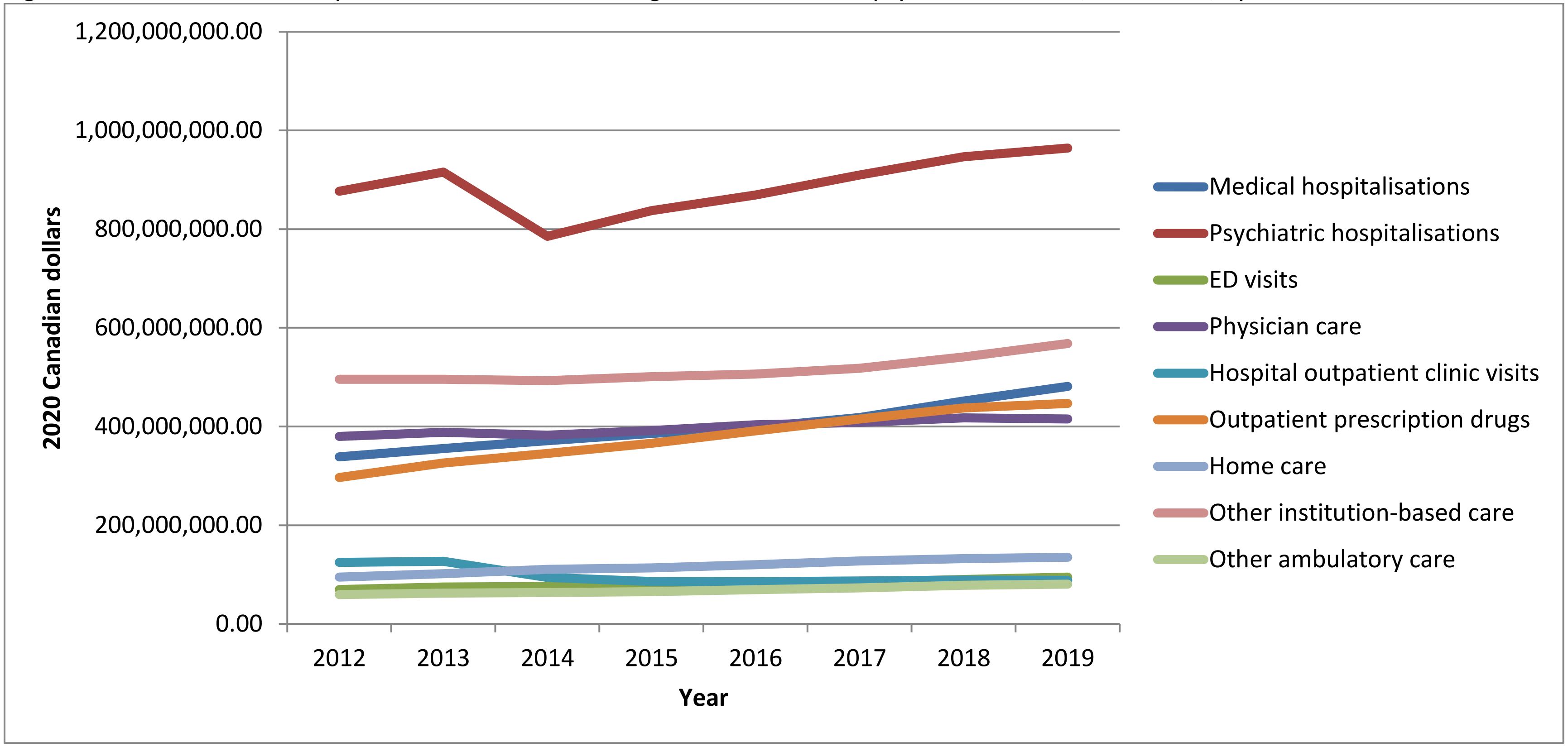

Source: administrative health care data from ICES

Figure A3. Mean number of psychiatric hospitalisations for individuals diagnosed with chronic psychotic disorders, 2012-2019, all and by sex

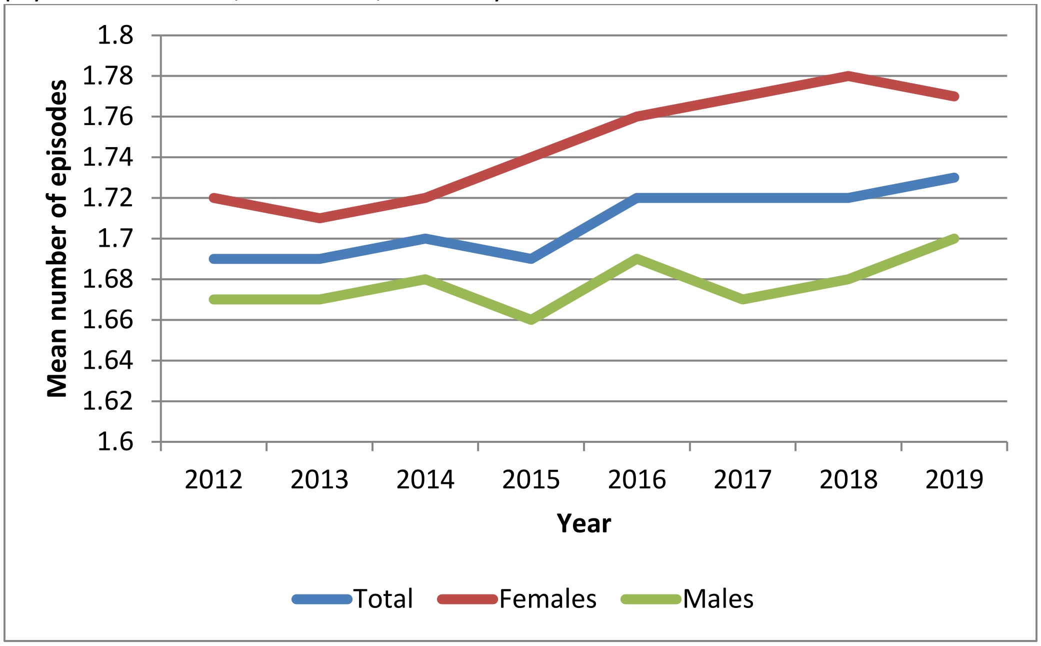

Source: administrative health care data from ICES

Figure A4. Mean length of stay of psychiatric hospitalisations for individuals diagnosed with chronic psychotic disorders, 2012-2019, all and by sex

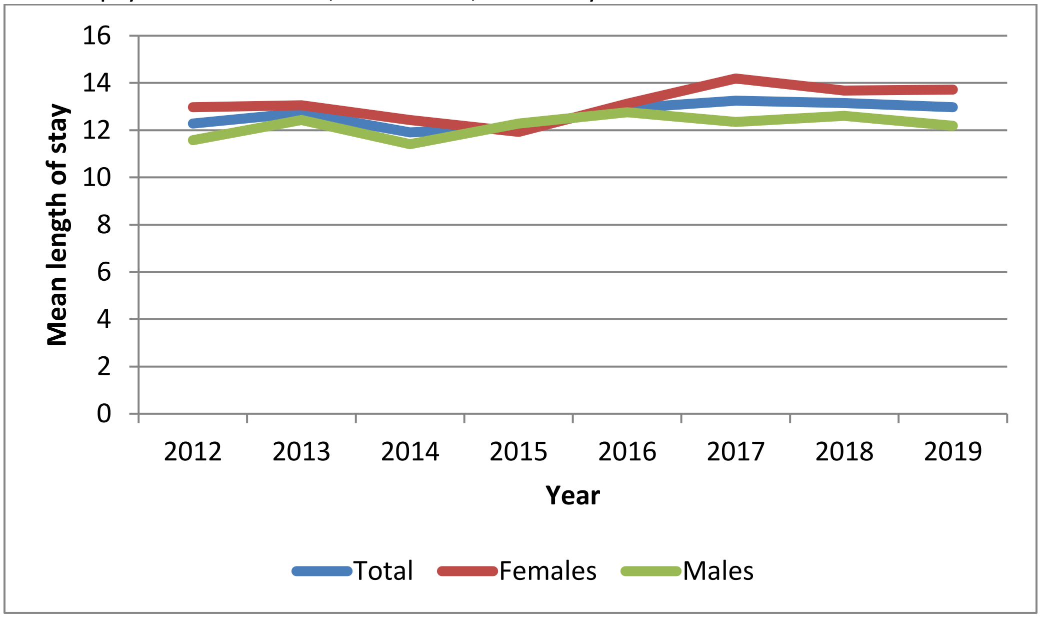

Source: administrative health care data from ICES

Figure A5. Mean number of acute medical hospitalisations for individuals diagnosed with chronic psychotic disorders, 2012-2019, all and by sex

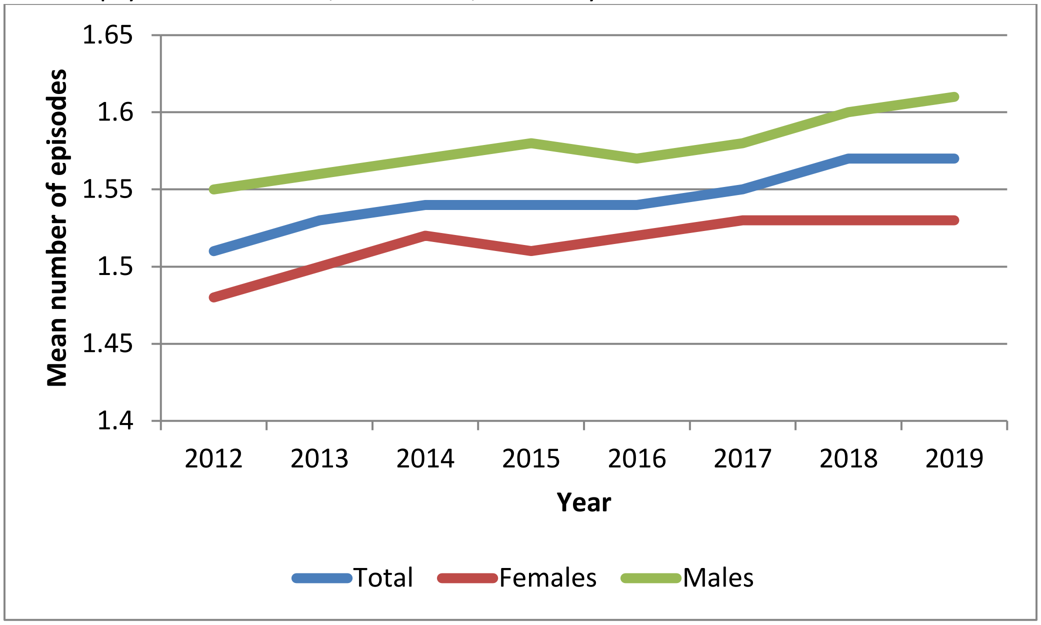

Source: administrative health care data from ICES

Figure A6. Mean length of stay of acute medical hospitalisations for individuals diagnosed with chronic psychotic disorders, 2012-2019, all and by sex

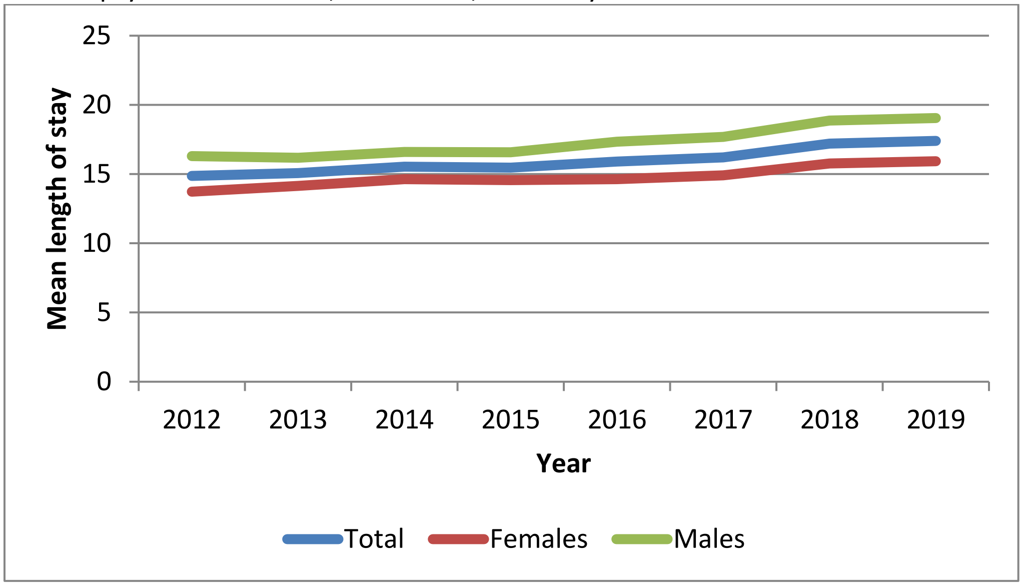

Source: administrative health care data from ICES

Figure A7. Mean number of comorbidities (measured using the Johns Hopkins Aggregated Diagnosis Groups software) for individuals diagnosed with chronic psychotic disorders, 2012-2019, all and by sex

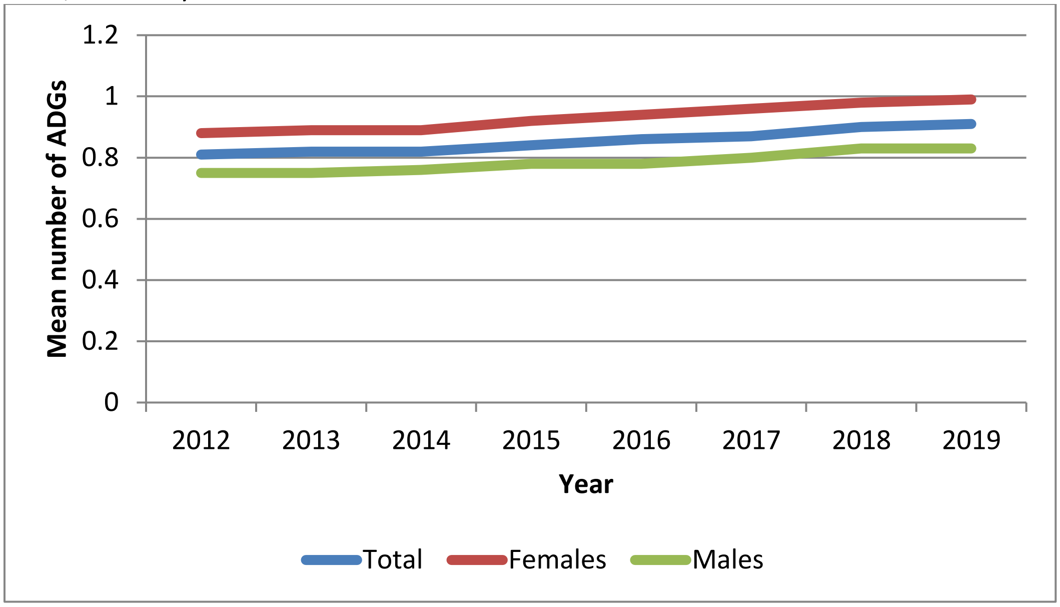

Source: administrative health care data from ICES

Figure A8. Mean number of chronic conditions for individuals diagnosed with chronic psychotic disorders, 2012-2019, all and by sex

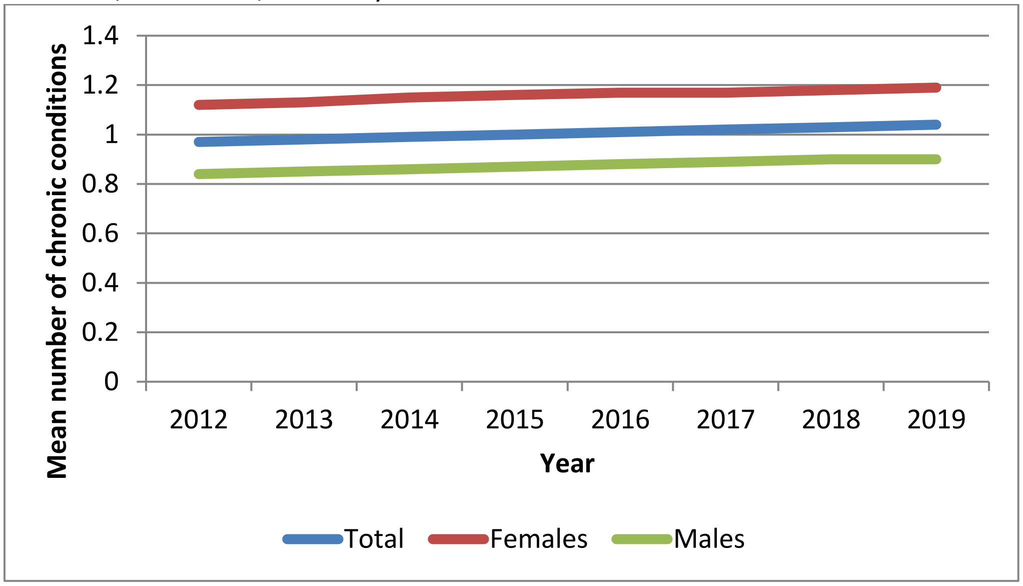

Source: administrative health care data from ICES

Figure A9. Total health care expenditures for individuals diagnosed with chronic psychotic disorders, 2012-2019, by number of comorbidities (measured using the Johns Hopkins Aggregated Diagnosis Groups software)

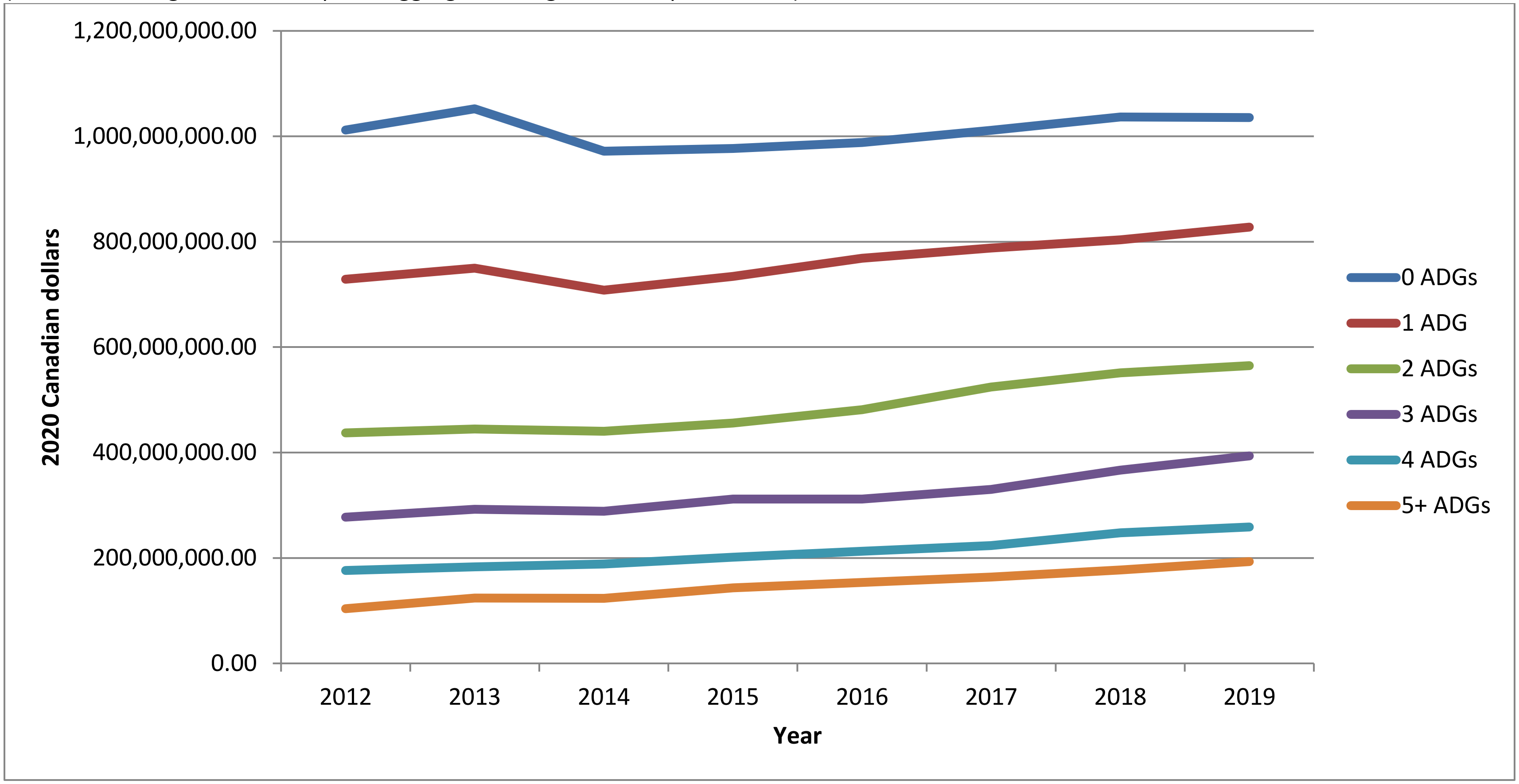

Source: administrative health care data from ICES

Figure A10. Total health care expenditures for individuals diagnosed with chronic psychotic disorders, 2012-2019, by chronic condition

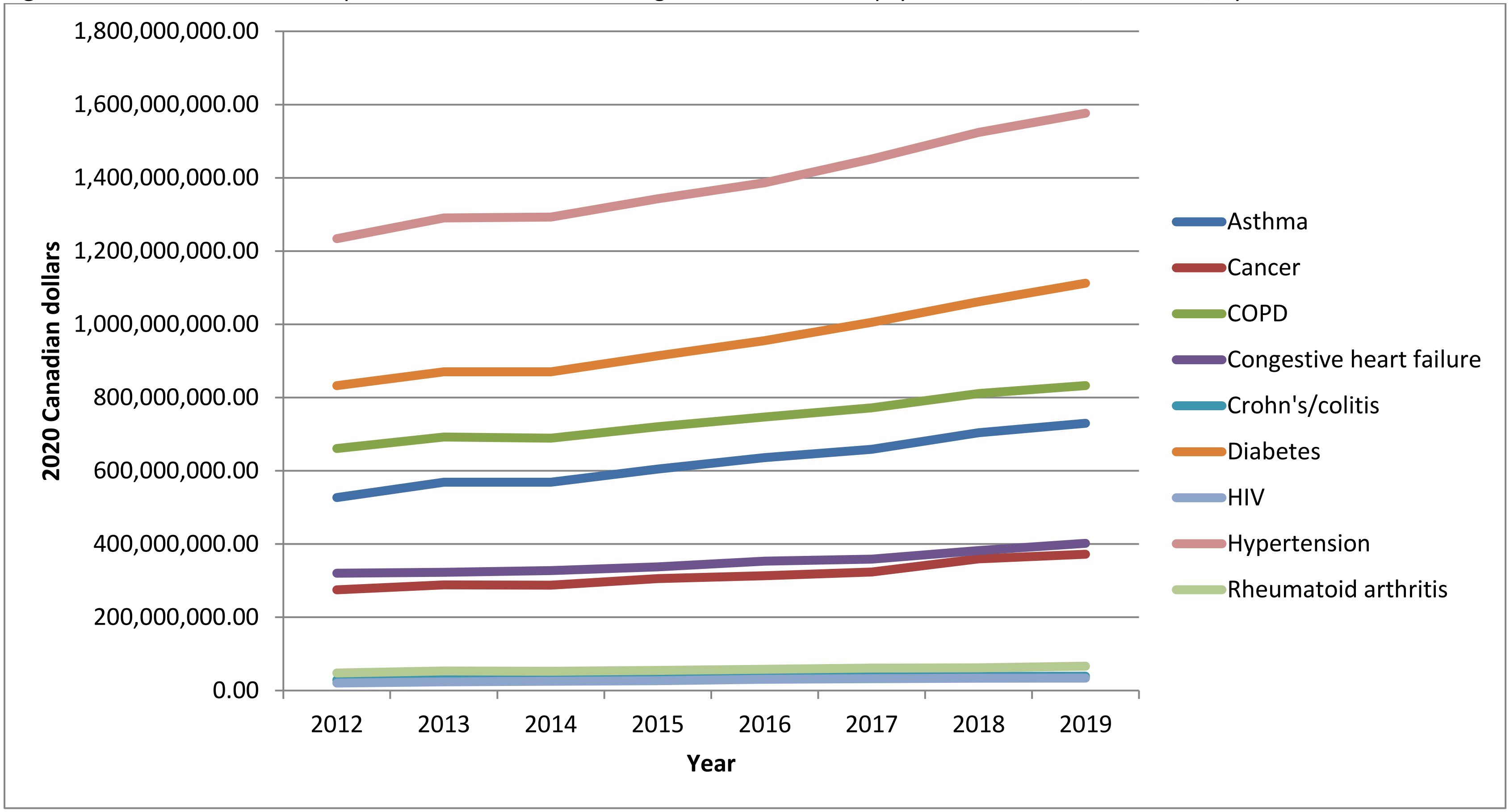

Source: administrative health care data from ICES
